# Random noise can help to improve synchronization of excimer laser pulses

**DOI:** 10.1098/rsos.150548

**Published:** 2016-02-10

**Authors:** Róbert Mingesz, Angéla Barna, Zoltán Gingl, János Mellár

**Affiliations:** 1 Department of Technical Informatics, University of Szeged, Árpád tér 2, Szeged 6720, Hungary; 2 Department of Experimental Physics, University of Szeged, Dóm tér 9, Szeged 6720, Hungary

**Keywords:** active timing control, jitter noise, dithering, programmable delay, excimer laser

## Abstract

Recently, we have reported on a compact microcontroller-based unit developed to accurately synchronize excimer laser pulses (Mingesz *et al.* 2012 *Fluct. Noise Lett.* 11, 1240007 (doi:10.1142/S021947751240007X)). We have shown that dithering based on random jitter noise plus pseudorandom numbers can be used in the digital control system to radically reduce the long-term drift of the laser pulse from the trigger and to improve the accuracy of the synchronization. In this update paper, we present our new experimental results obtained by the use of the delay-controller unit to tune the timing of a KrF excimer laser as an addition to our previous numerical simulation results. The hardware was interfaced to the laser using optical signal paths in order to reduce sensitivity to electromagnetic interference and the control algorithm tested by simulations was applied in the experiments. We have found that the system is able to reduce the delay uncertainty very close to the theoretical limit and performs well in real applications. The simple, compact and flexible system is universal enough to also be used in various multidisciplinary applications.

## 1. Introduction

Precise synchronization of events in time is essential in many experiments when time-dependent behaviour is monitored. Very short-time observations are often aided by impulse lasers in exploring many physical, chemical, biological phenomena, pump-probe measurements, and time-resolved spectroscopy [1–4]. A single trigger signal may generate multiple events including the laser shots and certain time delays appear at each individual component. Excimer laser operation itself may also need synchronizing units to control X-ray pre-ionization and master oscillator-power amplifier set-ups [5]. Lasers exhibit a varying time delay; therefore, this must be controlled in order to maintain measurement accuracy. The hydrogen thyratron used in excimer lasers to activate the high-voltage discharge has a certain switching time (so-called anode delay) which is subjected to a long-term drift caused by temperature changes. As a laser operated at high frequency is heated up, the anode delay may be changed by a few tens of nanoseconds. A random jitter is also present in laser systems due to the gas discharge uncertainty. This shot-to-shot jitter is not predictable; therefore, the slowly changing part only can be compensated. The proper control of the delay needs precise time measurement and tuneable delay units [6,7].

Rather surprisingly, the presence of random noise is not necessarily bad, sometimes adding noise can even improve performance. Today, there are several applications that are based on the constructive role of noise in various multidisciplinary fields. Examples include stochastic resonance that has special importance in biology [8–11] and dithering used in electrical engineering [12] and in image processing [13]. Recently, we have developed a universal, simple microcontroller-based delay-controller unit that features flexible software control and precise time-synchronizing hardware components [7]. Although the time resolution of the unit is limited to 10 ns, we could improve the timing accuracy well below this by using the jitter noise of the laser and additional random dithering. As a result stability close to the theoretical limit could be achieved. Up to now, the system's performance was tested by numerical simulations only; here we report on our experimental results of a laser pulse synchronization application in a KrF excimer laser.

## 2. Experimental set-up and delay-controller operation

**Figure 1.**
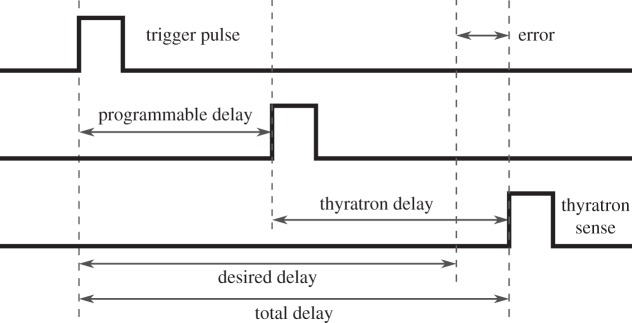
The principle of the control. The programmable delay can be used to compensate the changes in the thyratron delay and to achieve a total delay close to the desired value.

The principle of the control of the entire delay can be seen in figure 1. The rising edge of the external trigger impulse starts a programmable delay unit that activates the circuit of the thyratron. The high-voltage laser discharge is started by the thyratron signal and a laser pulse is generated. The desired delay is achieved by tuning the programmable delay; the total delay is the sum of this tuneable delay and the thyratron delay. The aim of the control is to minimize the error of the total delay, i.e. the difference between the total delay and the desired delay.

**Figure 2.**
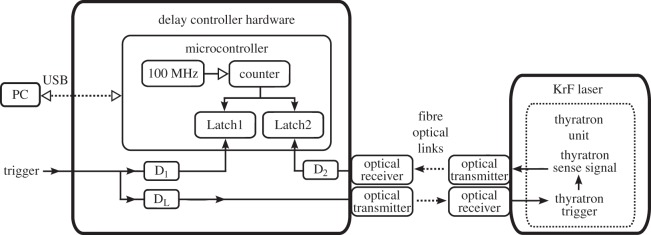
The block diagram of the experimental set-up.

In the following, we give a brief overview of the hardware components that were used to control the laser pulse timing. Note that only a smaller part of our more universal system was required; therefore, it is possible to build an optimized, even simpler unit in similar cases. The block diagram of the experimental set-up and the simplified hardware of the delay-controller unit is shown in figure 2.

**Figure 3.**
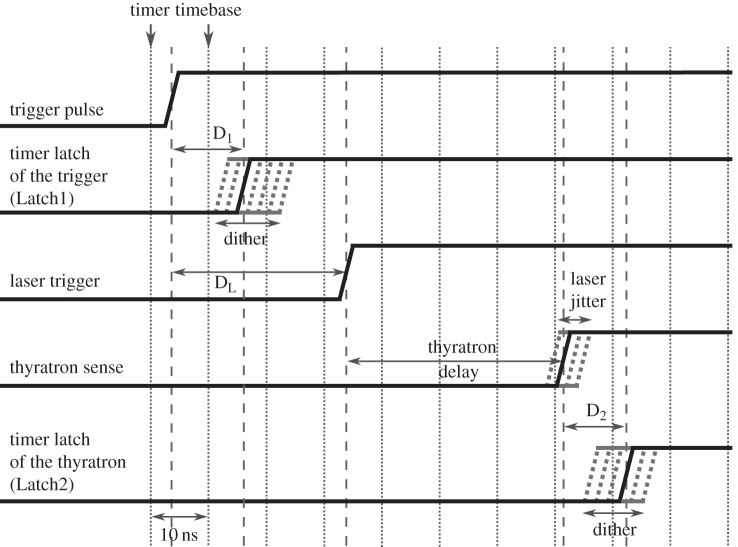
Time diagram of the delay control operation.

The delay-controller unit is based on a C8051F120 microcontroller incorporating a 100 MHz oscillator, a counter with two capture latches (Latch1 and Latch2) and a serial communication port. Three programmable delay units (D_1_,D_2_,D_L_) are integrated on the controller unit and the KrF excimer laser is connected to the rest of the system via fibre-optical links to reduce electromagnetic interference and noise. The whole system can be monitored by a host computer via a USB interface. The time diagram of the delay-control operation is shown in figure 3.

The programmable delay unit D_L_ can delay the triggering of the thyratron that activates the laser; this is used to achieve the desired overall delay. The delay time measurements are performed by time-to-digital converters implemented by the microcontroller's integrated programmable counter array. Its 16-bit main counter is driven by the 100 MHz oscillator in order to achieve a resolution of 10 ns. The two independent latches can capture the actual value of this counter upon the rising edge of the connected signals. The external trigger time instant delayed by the programmable delay unit D_1_ is measured by Latch1, while the thyratron sense signal delayed by D_2_ is captured by Latch2. The units D_1_ and D_2_ are required to optimize the quantization of the total delay time of the system by adding random dither to improve the resolution of the time measurement as was described in the externally triggered operation chapter of our initial article [7]. Subtracting the values stored in Latch2 and Latch1 gives the digitized total delay. As due to the dithering and laser jitter this value is fluctuating, adaptive averaging can be used to improve accuracy significantly; see the control algorithm section in [7]. The averaging time must be small enough in order to minimize the effect of the long-term drift of the thyratron delay. If the total delay is measured, it can be set to the desired value by tuning D_L_.

## 3. Experimental results

In order to reduce electromagnetic interference and to avoid possible ground loops the controller hardware and the KrF excimer laser trigger and thyratron sense signal were connected using fibre-optic cables, optical receivers (HFBR 2521) and transmitters (HFBR 1521). These elements introduce excessive jitter; therefore, first we evaluated the performance without the use of the KrF excimer laser. The desired delay was set to 1000 ns, and the jitter was measured using the time-to-digital conversion method described in the previous section. We found that the standard deviation of the jitter of the timing circuitry was 2.1 ns. In order to measure the full jitter of the system the delay control was switched off. The full delay between the laser trigger and the thyratron sense signals was measured to be 1.36 μs with a jitter of 10 ns. Owing to the statistical independence the full jitter is the root mean square of the laser jitter plus the jitter of the timing circuitry. In our case, this means that the contribution of the timing circuitry was below 2.2% of the full jitter. Lower jitter lasers may need even better performing optical receivers and transmitters. As a rule of thumb, the contribution to the total jitter can be kept below 5% if the jitter of the system is less than one-third of the laser jitter.

**Figure 4.**
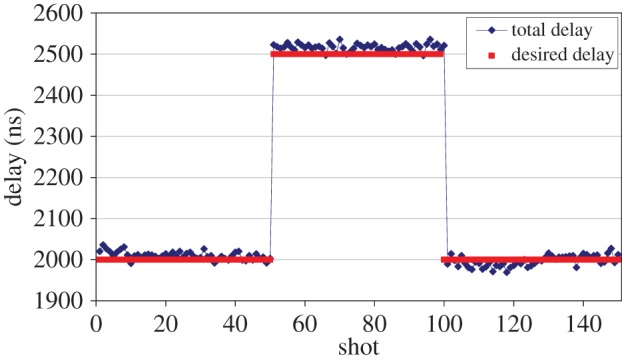
The regulated total delay and the desired delay as function of the laser shots.

After the testing the control circuit was tested with the KrF laser system. During the experiments, a pulse shape time-dependent desired delay was set as depicted in figure 4.

After 50 shots the initial value of 2 μs was changed to 2.5 μs and after the next 50 shots it was set again to 2 μs. Table 1 summarizes the measurement results for the three parts, in each case the number of averages was 50.

**Table 1.** Desired and measured delay and jitter for the three different parts of the laser delay regulation.

desired delay (μs)	measured delay (μs)	jitter (ns)
2.0	2.010±0.003	10
2.5	2.517±0.003	9
2.0	1.997±0.004	13

The measurement results show clearly that high accuracy can be achieved with the developed control system while the additional jitter due to the control system is negligible.

## 4. Conclusion

In addition to the results reported in our initial article, in this update paper we have demonstrated the performance of our compact microcontroller-based delay-control system driving a KrF excimer laser. We have added fibre-optic links to the original system to reduce its sensitivity to interference and noise, and we have shown that the time synchronization accuracy of the system can be maintained. Our experimental results confirm that the long-term drift of the laser delay can be removed by using the shot-to-shot jitter noise and added random dither with a simple and compact system. We have shown that the regulation can keep the accuracy close to the theoretical limit determined by the jitter of the laser. Lasers controlled by such system and algorithm can be used in many multidisciplinary applications where timing accuracy is important. Note also that the very compact and universal system can play a constructive role in general time-to-digital conversion and precision time synchronization solutions in various fields of science and engineering.

## Data accessibility

Data are available from the Dryad Digital Repository: http://dx.doi.org/10.5061/dryad.hg8m5.
